# Synthesis of organic aerogels with tailorable morphology and strength by controlled solvent swelling following Hansen solubility

**DOI:** 10.1038/s41598-018-19720-4

**Published:** 2018-02-01

**Authors:** Anurodh Tripathi, Gregory N. Parsons, Saad A. Khan, Orlando J. Rojas

**Affiliations:** 1 Department of Chemical and Biomolecular Engineering, North Carolina State University, Raleigh, NC 27695 USA; 2 Department of Bioproducts and Biosystems, School of Chemical Engineering and Department of Applied Physics, School of Science, Aalto University, FI-00076 Espoo, Finland

## Abstract

We introduce a generalized approach to synthesize aerogels that allows remarkable control over its mechanical properties. The Hansen solubility parameters are used to predict and regulate the swelling properties of the precursor gels and, consequently, to achieve aerogels with tailored density and mechanical properties. As a demonstration, crosslinked organogels were synthesized from cellulose esters to generate aerogels. By determination of Hansen’s Relative Energy Difference, it was possible to overcome the limitations of current approaches that solely rely on the choice of precursor polymer concentration to achieve a set of aerogel properties. Hence, from a given concentration, aerogels were produced in a range of mass densities, from 25 to 113 mg/cm^3^. Consequently, it was possible to tailor the stiffness, toughness and compressive strength of the aerogels, in the ranges between 14–340, 4–103 and 22–373 kPa, respectively. Additionally, unidirectional freeze-drying introduced pore alignment in aerogels with honeycomb morphologies and anisotropy. Interestingly, when the swelling of the polymeric gel was arrested in a non-equilibrium state, it was possible to gain additional control of the property space. The proposed method is a novel and generic solution to achieving full control of aerogel development, which up to now has been an intractable challenge.

## Introduction

Since the introduction of aerogels in 1931^1^, they have been synthesized from a variety of sources that include inorganic materials^2^, synthetic polymers^3^, biopolymers^4^ and carbons^5^; they have been recognized as promising systems for a plethora of applications, including membrane separation^6^, catalysis^7^, thermal and acoustic insulation^8^, flame resistance^9^ and sorption^10^. They are also used in fabrication of advanced materials such as supercapacitors^11^, cosmic dust collectors^12^, drug delivery devices^13^, photonics^14^, optics^15^ and mechanical energy absorbers^16^. Most of the applications cited require aerogels with high pore volume and low density along with high mechanical performance.

Aerogels are often produced from organogel precursors made by combining inorganic silica nanoparticles with suitable organic solvents. However these aerogels contain inherently weak interlinks within their structure that lead to overall poor mechanical performance^17^. Making aerogels from inorganic-organic composites has been offered as a solution for these limitations^18^. An attractive approach to make aerogels is the use of nano-materials such as nanocellulose^19^ and carbon nanotubes^20^, which are not solubilized but dispersed in appropriate media and have shown good mechanical stability, mainly due to presence of physical entanglements that provide ample contact points. However, chemical cross-linking is often needed for such nano-materials, as demonstrated by Yang *et al*.^21^, for cellulose nanocrystals, Zhang *et al*.^22^, for cellulose nano/micro fibrils and Zou *et al*.^23^, in case of carbon nanotube aerogels. Additionally, conventional polymers such as polyurea^3^ and polyimide^24,25^ have been used to form chemically cross-linked aerogels. These aerogels displayed impressive mechanical strength. However, despite such advancements, there is still a major need to systematically tailor the mechanical properties of polymeric aerogels. As a result, attempts have been made to control the density and pore volume of polymeric aerogels, mainly by changing the concentration of the precursor polymer^16,26–28^. However, this cannot be universally applied, and in some cases, it is not practical. For example, a low polymer concentration can result in aerogels of low density but display a limited number of chain-interactions, compromising the mechanical performance of the aerogel. Herein, we introduce a novel approach to address these issues: while keeping a given polymer concentration, the swelling state of precursor organogel is systematically controlled, which in turn defines the strength-to-weight ratio of the ensuing aerogel.

A crucial step in aerogel synthesis is the removal of the supporting fluid within the organogel without disrupting the network structure of the solid phase^29^. Various techniques are employed to produce the final gel, including supercritical drying, ambient drying and freeze drying. Materials produced by ambient drying and freeze drying are frequently called xerogels and cryogels, respectively. However, materials with pore volume above 90%, synthesized by any of these process are often termed as “aerogels”^29^. For simplicity, we use the term “aerogel” for all materials prepared in this study.

During aerogel synthesis, solvent exchange is usually done to facilitate solvent removal. For example, in typical supercritical drying, the fluid in the gel is replaced with a transitional solvent, followed by an exchange with supercritical CO_2_ in a high-pressure chamber. The supercritical CO_2_ is then vented out at critical point conditions (31.1 °C, 72.9 bar) thereby yielding the final aerogel^30^. Likewise, solvent exchange is useful for ambient drying, where a low surface energy solvent is introduced to reduce capillary forces during solvent evaporation^31^. On the contrary, reports using solvent exchange for the aerogels produced via freeze-drying are not readily found.

We hypothesize that solvent exchange for the aerogels produced during freeze-drying can provide novel routes to create aerogels with a better control over their mechanical properties. The solvent exchange leads to either gel swelling or shrinking. Systematically choosing the exchange solvent and conditions can control the organogel volume change. By controlling organogel swelling and shrinkage, we can tailor the density of the resulting aerogel and consequently, its mechanical properties.

The main premise of this study is to understand the involved phenomenon that can facilitate the prediction and control of swelling/shrinking behavior of the precursor organogel. Thus, we performed a systematic analysis based on the Hansen solubility parameter, relevant to swelling and shrinkage of organogels during solvent exchange. The Hansen solubility parameter approach is a relatively simple approach that accounts for polar and hydrogen bonding interactions, which are very common in the polymers of interest. In addition, the Hansen solubility parameter approach affords prediction capability in multicomponent systems^32^. It is noted that recently Hansen solubility parameters have been expanded to investigate particle dispersions such as carbon nanotubes^33^, cellulose nanocrystals^34^, clays^35^ and others. However, in these cases modification is needed for the model to be successfully implemented it these and related systems. Therefore, we have limited our study to fully solubilized polymers, which are appropriate in the framework of the conventional Hansen solubility parameter model.

As a proof-of-concept, we explore this method using cellulose acetate. Cellulose acetate is a biopolymer, derived from cellulose, that exhibits polar and hydrogen bonding interactions. It also displays good wet strength, which is a great limitation for traditional cellulosic materials^36^. The very limited work available on cellulose acetate aerogels reports low pore volume (41%) and high density (0.85 g/cm^3^) materials after synthesis via supercritical drying^27,37^. In our previous work, we synthesized ultra-light aerogels from cellulose acetate of density as low as 0.004 g/cm^3^^38^. Herein, we build on that study to introduce a simple, systematic and general approach based on the selection of solvency (as defined by the solubility parameter) to tune swelling state of the resulting organogel thereby providing a means to control morphology and properties of the as-produced organic aerogel product.

Therefore, through this study, we uniquely demonstrate that combining rational solvent exchange with freeze-drying can enable synthesis of aerogels with more favorable properties than those made by more traditional routes.

## Results and Discussion

For this study, organogels are formed using cellulose acetate (CA) in acetone, followed by solvent exchange in a mixture of acetone and water and freeze-drying to achieve the final aerogel product. To understand the effect of the mixture of acetone-water solvent on the organogel during solvent exchange, we first describe how the Hansen solubility parameters affect the swelling of cellulose acetate. Following that analysis, we discuss the effect of swelling/shrinkage on the morphological and mechanical properties of the resulting aerogels, including an analysis of the formation of freeze-dried aerogels via unidirectional freezing to isolate the effect of freezing on the mechanical properties of the aerogel. Finally, we elaborate on the role of solvent exchange on aerogel properties by evaluating the effect of arresting the organogel swelling in a non-equilibrium state during solvent exchange.

### Hansen solubility parameters and swelling

The Hansen solubility parameters provide a quantifiable guide to determine solubility of one material into another. In this study, we use the Hansen solubility method to understand solubility of cellulose acetate (CA), with acetyl content of 39.7%, in acetone/water solvent mixtures.

A general concept of Hansen solubility parameter in terms of the Hansen solubility sphere (Figure S1) is described in the supporting information. Briefly, the Hansen solubility parameter accounts for dispersion (*δ*_*d*_), the polar (*δ*_*p*_) and hydrogen bonding (*δ*_*h*_) that arise, respectively, from van der Waals, dipole and hydrogen bonding interactions. These three components, the Hansen solubility parameters (HSP), can be factored in or calculated as *Ra*, Equation (1):

1 $$Ra=\sqrt{4(({\delta }_{d1}^{2}-{\delta }_{d2}^{2})+({\delta }_{p1}^{2}-{\delta }_{p2}^{2})+({\delta }_{h1}^{2}-{\delta }_{h2}^{2}))},$$

where, *Ra* is the difference between the HSP of a solvent (1) and a polymer (2). The constant 4 is from an empirical correlation. The solubility of the polymer in a solvent maintained if *Ra* < *R*_0_, where *R*_0_ is defined as the “interaction radius” of the polymer, which is measured experimentally^32^. A Relative Energy Difference (RED) is defined as *Ra/R*_0_. A value of RED < 1 implies good solubility for the polymer in the given solvent.

For cellulose acetate in acetone/water mixtures, the values of the respective HSPs and *R*_0_ of the polymer is displayed in Table 1. The values for CA, water and acetone are determined experimentally elsewhere^32^.

**Table 1:** Hansen solubility parameters (HSP) corresponding to the polymer (cellulose acetate, CA) and the solvents (mixtures of water and CA)^32^. AVF is the Acetone Volume Fraction in water.

	*δ*_*d*_ (MPa)^1/2^	*δ*_*p*_ (MPa)^1/2^	*δ*_*h*_ (MPa)^1/2^	*R*_*0*_ (MPa)^1/2^
CA	18.6	12.7	11.0	7.6
1.0 AVF (Acetone)	15.5	10.4	7.0	—
0.9 AVF	15.5	10.9	10.2	—
0.75 AVF	15.5	11.8	15.8	—
0.5 AVF	15.6	13.2	24.7	—
0.25 AVF	15.6	14.6	33.5	—
0 AVF (Water)	15.6	16.0	42.3	—

**Figure 1:**
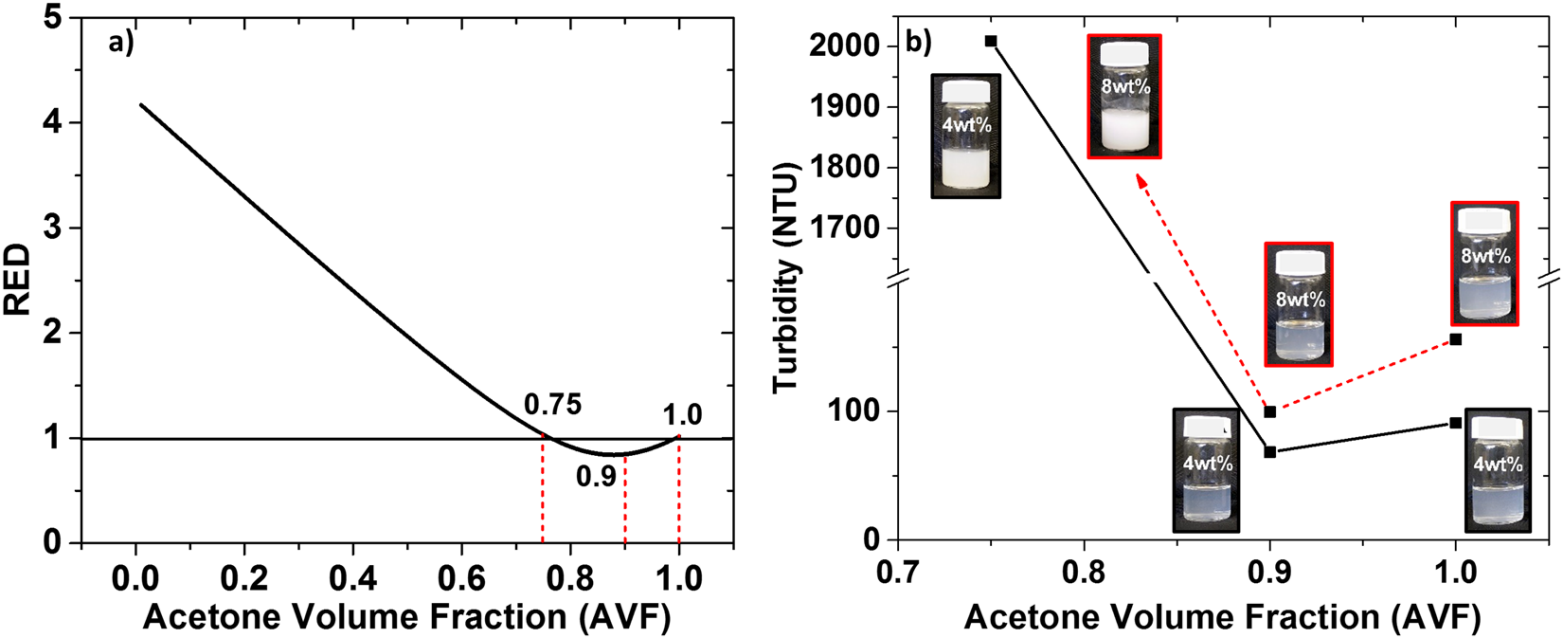
(**a**) Relative energy difference (RED) between CA and the solvent blend of acetone and water. (**b**) Turbidity measurement of the 4 and 8 wt% CA solutions in acetone/water solvent. The turbidity (NTU) value for 8 wt% CA in 0.75 AVF was out of the instrument limit, i.e., 10,000NTU. Images show solution of CA in solvent blends with of different AVFs, represented in the image. Note a drastic increase in turbidly in water-rich solvents, at AVFs of <90%.

The HSPs of the solvent blend consisting of small molecules, such as acetone and water, can be calculated by simple rule of mixing. Figure 1a shows the RED as a function of acetone volume fraction (AVF) in the solvent blend. It is evident from the graph that the blend with 0.9 AVF is theoretically the best solvent for CA. This claim is corroborated experimentally from turbidity analysis of the CA solutions (4 and 8 wt%) in solvent blends of AVF 1, 0.9 and 0.75. As can be seen from Fig. 1b, the CA solution in 0.9 AVF exhibits the lowest turbidity. The corresponding images of the solutions are also shown in Fig. 1b.

This understanding of the behavior of CA polymer in the solvent blend of acetone/water can be used to tune the swelling behavior of CA gels. The analysis indicates that CA gel must exhibit maximum swelling in the best solvent (0.9 AVF) due to relaxation of the polymer chains. As can be seen in Table S1, the polar and hydrogen bonding component is critical in defining the interaction between the solvent and CA. In addition to a minimum value of the polar component (>5 MPa^1/2^), the hydrogen bonding component of the solvent must be similar to that of CA. Therefore, on addition of 10% water, the hydrogen bonding component of the blend becomes similar to that of CA, allowing the 10% blend (AVF = 0.9) to become ‘‘better’’ solvent for CA.

**Figure 2:**
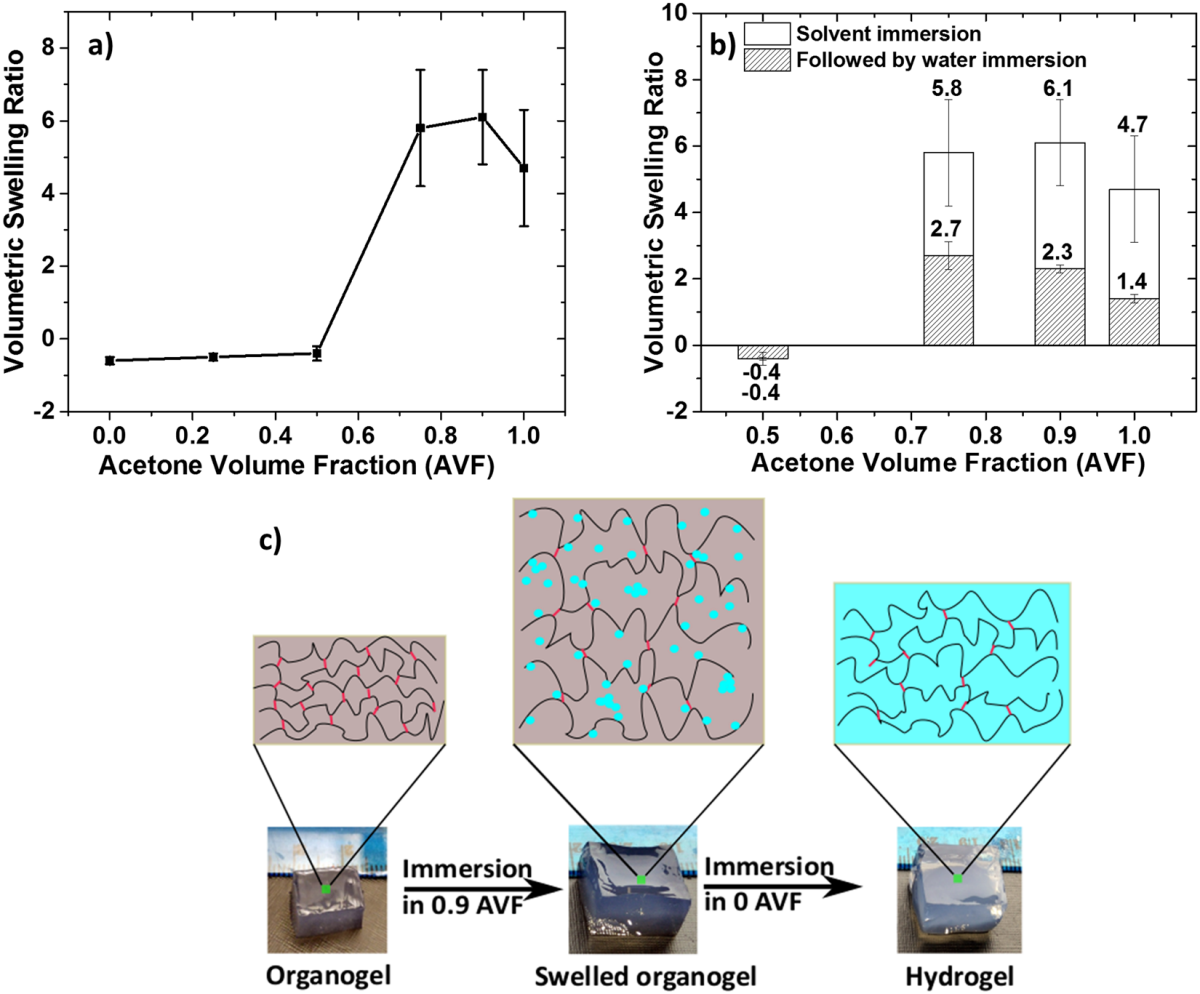
(**a**) Swelling behavior of cross-linked organogels as a function of the acetone volume fraction (AVF) in the solvent. (**b**) Equilibrium volumetric swelling ratio after exchange with water. (**c**) Schematic illustration of the swelling of cross-linked CA gels (color code: black chains-CA polymer; red dash- cross-links; blue dots-water molecules; grey- acetone and blue- water). Hydrolysis of ester bonds is expected when the organogel comes in contact with water, resulting in irreversible swelling of the gel, termed here “hydrogel” (from the swelled organogel to the hydrogel, as indicated by the size of the image).

The CA gel or organogel was formed by dissolving CA in anhydrous acetone along with the pyromellitic dianhydride cross-linker. The cross-linking reaction was catalyzed by triethylamine. Note that the swelling of gels also depends on the cross-linking density. Here, the cross-linking density was kept constant to isolate the effect of solvent composition (see experimental section for details). The CA gels were cut in cuboidal shapes and immersed in the mixture of acetone and water with known AVF. The volumetric swelling ratio was measured, and result are given in Fig. 2a. The maximum swelling predicted for CA organogel from HSP is at 0.9 AVF. However, the swelling exhibits a maximum at AVF between 0.75 and 0.9. The observed range of AVF instead of a fixed value, as predicted from HSP, is most likely due to the chemical cross-linking of CA, which may cause a slight shift in the “good solvent” concentration for CA organogels. As expected from the Hansen solubility parameters, solvent blends below 0.5 AVF are poor solvents for CA and they do not show any appreciable swelling.

The obtained organogels were then immersed in DI water for 72 h and the DI water was replaced two times in between to remove excess acetone. The as obtained gels are termed hydrogels. Interestingly, even after immersing the swelled organogels in the poor solvent (water), they did not shrink back to the original state; in fact, they equilibrated at a given final swelling ratio, Fig. 2b. The final swelling ratio ranges from 2.7 to 1.4 for AVF of 0.75 to 1. The organogel in 0.5 AVF did not show any further shrinkage after immersing in water. The fact that the swelled organogel did not revert to its original volume or shrunk further when immersed in a poor solvent can be explained by the elastic behavior of the cross-linked networks and presence of hydroxyl groups on cellulose backbone.

We hypothesize that the elastic network behaves as illustrated schematically in Fig. 2c. The chains drawn in solid lines denote the CA polymer while the lighter, red lines denote cross-links. The (blue) dots represent water molecules and the grey and blue represent acetone and water respectively. Immersing organogel in a good solvent blend (0.9 AVF, middle image in Fig. 2c) causes swelling and allows water molecules to seep into the network along with acetone. These water molecules may hydrolyze some of the ester bonds, thus reducing the elasticity of the cross-linked network^39^. Upon immersing swelled organogels in a poor solvent (AVF = 0, right image in Fig. 2c) for a certain period, the acetone molecules diffuse out while the water molecules remain trapped inside, due to hydrogen bonding interactions with the hydroxyl groups of CA polymer chains.

The hydrolysis of ester bonds should cause weakening of the elastic network that can be qualitatively correlated with lowering of elastic modulus (G’) of the resulting hydrogel. To test this hypothesis, three hydrogel samples were prepared by immersing the organogel for 4, 12 and 24 h in solvent of AVF = 0.9 followed by immersion in water for 72 h and rheological testing. The rheology tests involved performing a frequency sweep (1–100 rad/s) on the resulting hydrogels using a serrated flat plate geometry. This test provides values of G’ which is the measure of elastic modulus of the hydrogel. If the hydrolysis is ester bonds is occurring, then it is predicted that organogel immersed for longer period (24 h) will have the lowest elastic modulus (G’). The result shown in Figure S2, indicates a three-fold decrease in G’ upon increasing the immersion time for the gel, from 4 to 24 h, thereby lending credence to our premise.

It should be noted that even though cellulose acetate was chosen as the model polymer, the illustrated concept of controlling polymer swelling via Hanson Solubility Parameter analysis can be easily extended to other polymers. Upon identification of the interaction radius R_0_ of the polymer, suitable solvents or solvent mixtures can be chosen based on their solubility parameter (δ_d_, δ_p_, δ_H_), that either swell or shrink the gel relative to their position on the Hansen sphere. For example, Diehn *et al*.^40^, used HSP approach to predict *a priori*, the self-assembly of 1, 3, 2, 4-dibenzylidene sorbitol in presence of various solvents with different HSPs and their distance from R_0_.

### Aerogel Synthesis and properties

**Figure 3:**
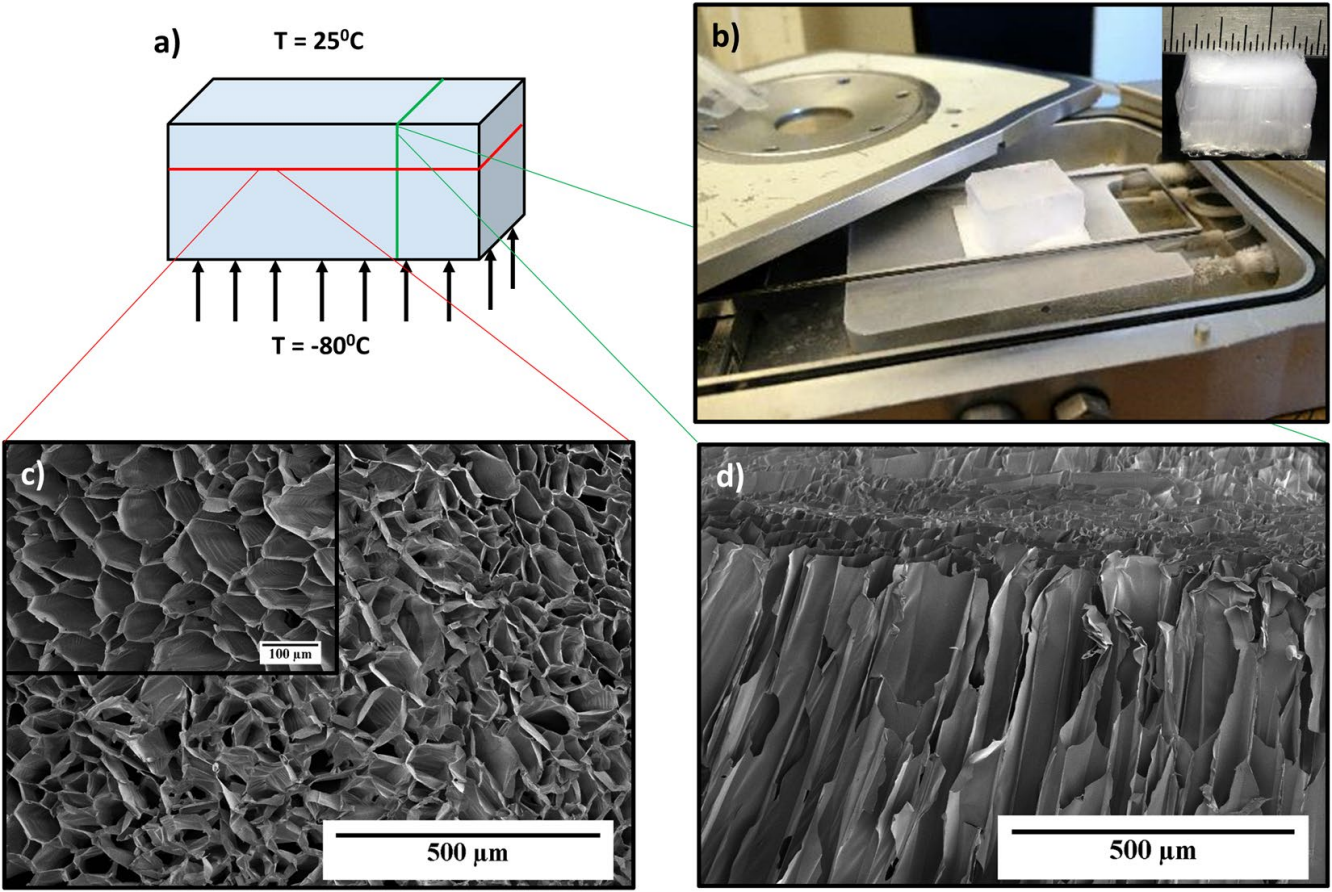
(**a**) Illustration of unidirectional freezing of hydrogel. (**b**) Image showing the unidirectional freezing arrangement. Inset shows the CA aerogel, (**c**) SEM image of out-of-plane view (cross-section perpendicular to the freezing direction). Inset shows the magnified SEM image (scale: 100 μm) and, (**d**) SEM image of in-plane view (cross-section in direction to the freezing direction).

For our studies, we use nomenclature to identify the aerogels produced in different solvent exchange conditions. For example, an aerogel obtained from the gel that was solvent-exchanged in AVF = 0.9 is referred to as “0*.9* *A*”. In general, it can be expected that the mechanical properties of such aerogels depend on the pore structure that includes pore volume and alignment as well as inherent wall thickness. It has been shown that that freezing conditions strongly affects the pore structure of the resulting aerogel^41,42^. Therefore, to isolate the effect of freezing conditions on the mechanical properties of the aerogels, unidirectional freezing was performed to produce uniform pore alignment and structure. The procedure and setup is shown in Fig. 3a,b. The hydrogel was placed on a copper plate that was kept at a constant temperature of −80 °C. The other faces of the hydrogel were kept open to atmosphere at 25 °C. The frozen hydrogel was freeze dried at −56 °C and 0.113 mbar, to give the aerogel as shown in the inset of Fig. 3b. The out-of-plane (cross-section perpendicular to the freezing direction) and in-plane (cross-section along the freezing direction) view of SEM micrographs of CA aerogels are presented in Fig. 3c,d, respectively. The out-of-plane view of CA aerogel (Fig. 3c) exhibits a honey-comb structure with cell sizes ranging from 50 to 100 μm. The closed pore structure resembles a morphology that is reminiscent of sublimated ice crystals. Since the growth rate of ice crystals is highly anisotropic in one direction, the CA polymer is forced to align along the solidification front. The CA polymer becomes concentrated and squeezed on to the crystal boundaries giving the shown highly ordered honeycomb structure. The inset in Fig. 3c shows the magnified SEM image of the out-of-plane view. The in-plane view, Fig. 3d, exhibits a directionality in the pores. Most of the CA polymer was aligned in the direction of ice crystal growth and no honeycomb structure was observed in this view. Similar pore alignment with honeycomb structure has been observed for directional freezing of cellulose nanowhisker foams^43^ and a composite foam based on nanocellulose and graphene oxide^44^. However, the pore structure is not limited to columnar geometries. Chau *et al*.^45^, for example, demonstrated fibrillar and lamellar morphology, in addition of honeycomb-like columnar systems, during directional freezing of hydrazone crosslinked CNC/POEGMA aerogels.

It should be noted that the immersion of CA gel into water (i.e. a poor solvent according to HSP) may cause phase separation, leading to hierarchical porosity with pore sizes of <1 μm. However, results show only large pores, 50–100 μm in size, formed between the non-porous walls. We believe that phase separated structures of CA, if any, were eliminated during the freezing step, possibly by being compressed into thin walls of the polymer during slow freezing of water. To confirm this idea, we performed cryo-SEM of a hydrogel prepared from solvent exchange in AVF = 0.9. During sample preparation for cryo-SEM, the hydrogel was frozen rapidly in liquid nitrogen to prevent sample destruction by volume expansion of ice. This was followed by sublimation of ice, coating with gold and imaging of the sample. The obtained SEM image shown in Figure S3a,b demonstrate CA arrangement in honey-comb patterns, as observed before. Magnification of a small section of Figure S3b, where sample destruction was prevented by rapid freezing, indicates a phase-separated CA polymer structure (Figure S3c), as predicted. The final properties of the dried aerogels, however, are mainly affected by their bulk properties, which are governed by the macroporous structure obtained upon unidirectional freezing of the hydrogel.

**Figure 4:**
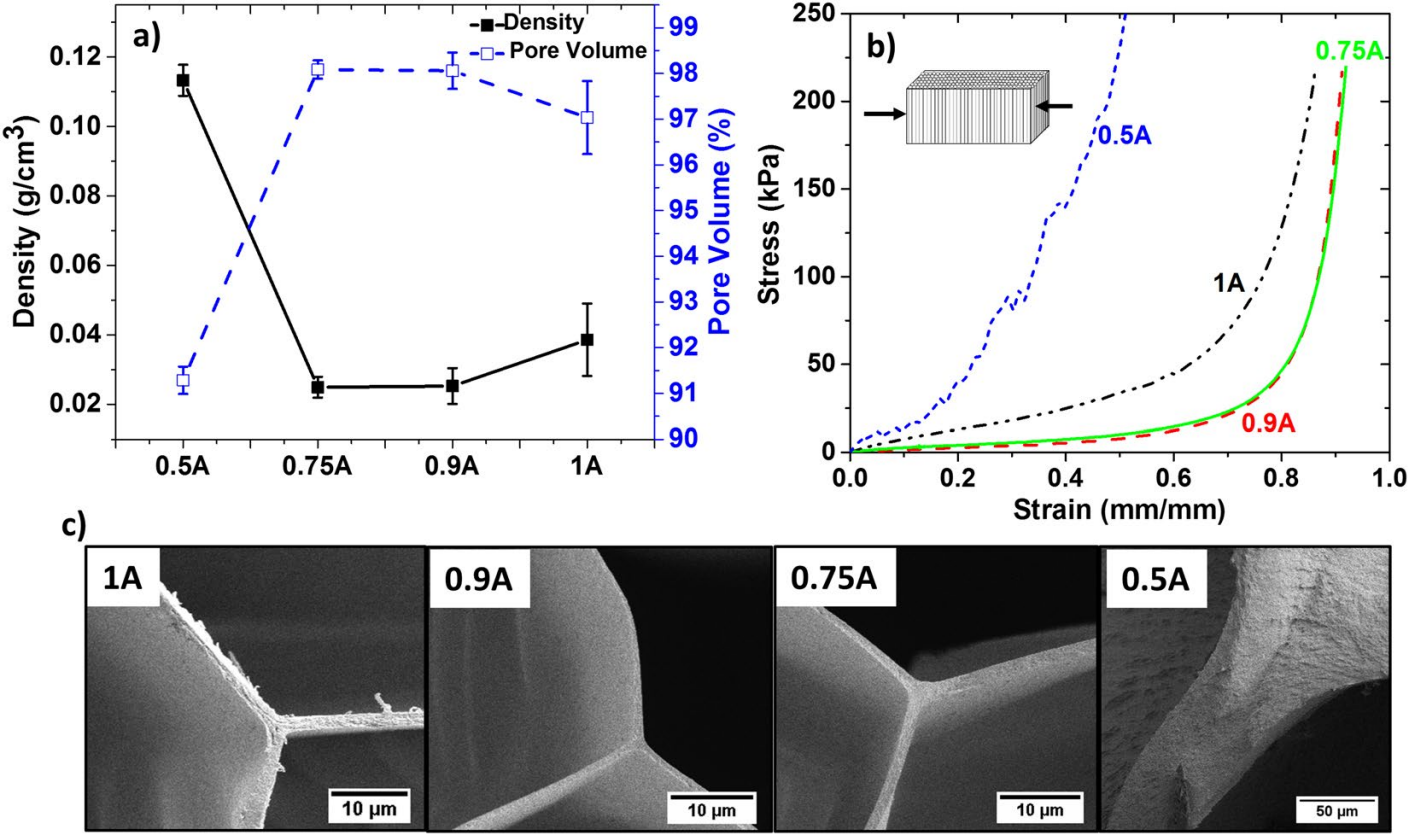
(**a**) Density and pore volume (%) of CA aerogels synthesized from hydrogels that were obtained after solvent exchange with different acetone volume fractions (AVF). (**b**) Compressive stress-strain curve for the CA aerogels. Illustration in the inset shows the compression direction (out-of-plane compression). (**c**) SEM images of the pore wall of the aerogels. The wall thickness of aerogels is corresponding to 1.3 ± 0.3 µm (*1* *A*), 0.8 ± 0.1 µm (*0.9* *A*), 0.8 ± 0.1 µm (*0.75* *A*) and 40 ± 12 µm (*0.5* *A*).

The CA aerogels produced by solvent exchange and freeze drying were analyzed for their structural and mechanical properties. As seen from the Fig. 4a, aerogels *0.9* A and *0.75* A have minimum density and highest pore volume. The 4 wt% CA hydrogel undergoes minimal shrinkage during freeze drying. Hence, the difference in densities and porosities is attributed to the swelling behavior of CA organogels when immersed in solvents with various AVF. The organogels immersed in 0.9 and 0.75 AVF exhibited maximum swelling, resulting in aerogels with lowest densities and highest pore volume. The organogel immersed in 0.5 AVF (poor solvent for CA) did not exhibit swelling, which resulted in aggregation of the polymer chains even before freezing, thus producing an aerogel with highest density and lowest pore volume. Figure S4 shows SEM images of the aerogels *1A-0.5* *A*. The aerogels *1* *A, 0.9* *A and 0.75* *A* exhibit a honeycomb pattern that spans the entire aerogel. The aerogel *0.5* *A*, on the other hand, shows pores that do not span the entire aerogel.

The mechanical properties of the aerogel were analyzed by measuring the stress as a function of compression strain. Figure 4b shows the out-of-plane compressive stress-strain curve for the CA aerogels, with the inset demonstrating the experimental schematic. The curves resemble compression curves of elastomeric foams, as introduced by Gibson and Ashby^46^. The elastomeric foams are characterized by a linear elastic region followed by a plateau region and a final densification region. The linear elastic region is controlled by cell wall bending. A small linear elastic region is observed (less than 3% strain) for CA aerogels, which is due to low relative density of the aerogels (<0.09). The aerogels *1* *A, 0.9* *A* and *0.75* *A* have extremely low relative density (0.03–0.02), which indicates the presence of thin cell walls and edges, as seen in Fig. 4c. The cell wall thickness of aerogels *1* A, *0.9* *A* and *0.75* *A* corresponds to 1.3 ± 0.3, 0.8 ± 0.1 and 0.8 ± 0.1 μm, respectively. Furthermore, a honeycomb pattern of the thin cell walls (Figure S4) induces a large pore volume and provides an added strength to the aerogel.

The linear elastic region is followed by an extended plateau region (for aerogels *1* *A, 0.9* *A* and *0.75* *A*, up to 70% strain), which is associated with collapse of the cells walls by elastic buckling. The arrangement of thin cell walls in a honeycomb pattern gives an excellent load bearing capacity to the aerogel structure and allows aerogels *1A-0.75* *A* to be compressed to large strains of more than 80%. A cyclic compression and release for *0.9* *A* aerogels upon increasing strain is shown in Figure S5. This indicates that the *0.9* *A* aerogel maintains its mechanical integrity until 50% strain but a permanent deformation is achieved at 80% strain. Furthermore, the same *0.9* *A* aerogel, after being subjected to 80% compression cycles, demonstrated an excellent elasticity, when compressed repeatedly, out-of-plane (Movie S1). This is most likely because the structure reached an equilibrium state after initial collapse and permanent bending of some cell walls. The aerogel *0.5* *A*, in contrast, has a high relative density (0.09), corresponding to the observed thick cell walls and edges (40 ± 12 μm). In addition, the honeycomb structure of aerogel *0.5* *A* did not span the entire system (Figure S4), which generates structural defects exhibited in the form of kinks on the stress-strain curve.

When the cells are completely collapsed, further strain causes the cell walls to touch each other resulting in a sudden rise of stress. This region of densification is very distinct for aerogel *1A-0.75* *A* due to a large pore volume. In contrast, aerogel *0.5* *A* exhibits a short plateau region that transitions quickly to the densification region, as explained by its low pore volume.

Generally, the mechanical properties of an aerogel such as compression modulus, absorption energy, compressive strength and densification strain is highly dependent on its relative density^46^. Usually, aerogel density is tailored by varying the polymer content. However, reducing the polymer content compromises mechanical performance of the aerogels. Significantly, in this study, the density was tuned by controlling the swelling behavior of the precursor CA organogel.

Using the controlled swelling approach developed here, we find that the density and desired mechanical properties of the aerogels be tailored, based on the basic understanding of swelling phenomenon. Moreover, we find that the mechanical integrity of the aerogels can be favorably maintained. As can be seen from Table 2, the resulting aerogels show a wide range of values for aerogel stiffness (compression modulus from 14 to 340 kPa), toughness (absorption energy from 4 to 103 kPa) and strength (compressive strength from 22 to 373 kPa). A wide range of densification strain (35 to 87%) was also observed. The densification strain gives an indication to the compressibility of an aerogel that arises from the large pore volume and elastic pore walls arranged in the observed honeycomb pattern. The values imply the flexibility of the proposed solvent exchange approach used to synthesize aerogels with a range of comprehensive mechanical properties without undergoing any chemical or physical modification.

**Table 2:** Mechanical properties of the CA aerogels.

	*1 A*	*0.9 A*	*0.75 A*	*0.5 A*
Density, ρ_a_ (g/cm^3^)	0.039	0.025	0.026	0.113
Pore Volume (%)	97.0	98.1	98.0	91.3
Relative density (ρ_a_/ρ_CA_)	0.030	0.019	0.020	0.087
Compression Modulus (kPa)	110	14	32	340
Absorption Energy (kPa)	18	4	6	103
Compressive strength (kPa)	71	22	24	373
Densification strain (%)	78	86	87	35

### Introducing Anisotropy

**Figure 5:**
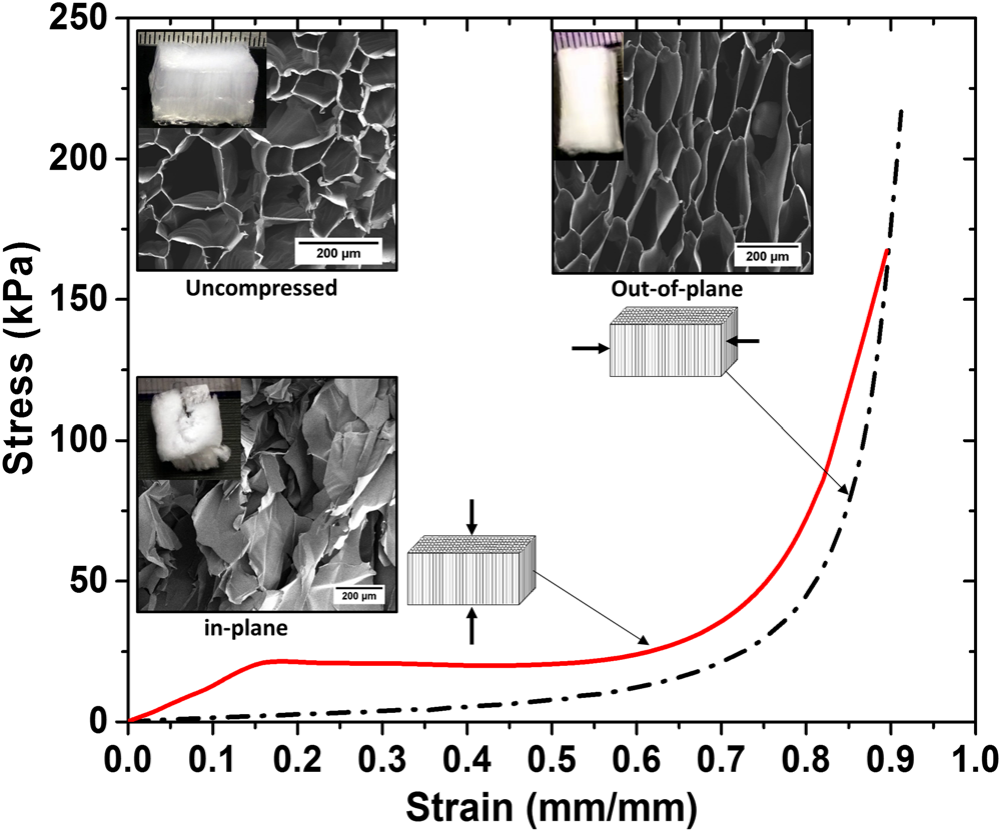
Compressive stress vs strain curves for aerogel *0.9* *A* when compressed in-plane and out-of-plane. Inset shows out-of-plane SEM micrographs of the aerogel *0.9* *A* when, uncompressed (top left), compressed out-of-plane (bottom left) and compressed in-plane (top right). Photos of aerogel samples are also shown.

**Figure 6:**
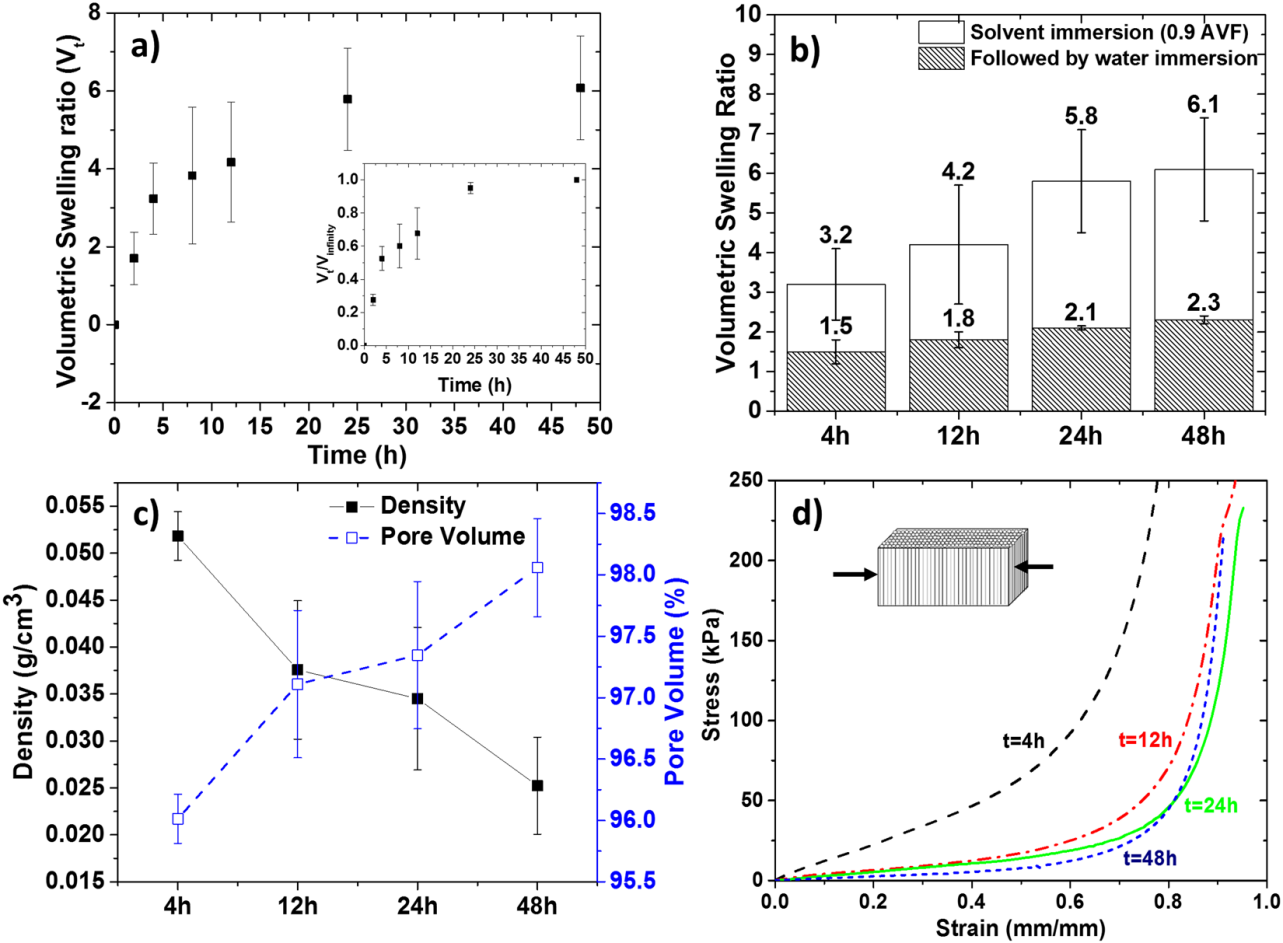
(**a**) Kinetics of volumetric swelling ratio for organogel immersed in 0.9 AVF. Inset has swelling ratio normalized with maximum swelling observed at 48 h. (**b**) Swelling ratio measured after immersing in water for 72 h. (**c**) Density and pore volume of the aerogels synthesized after varying the solvent exchange time. (**d**) Compressive stress vs strain curves for the aerogels obtained after varying solvent exchange time.

The pore alignment of the aerogels due to unidirectional freezing endows an anisotropic behavior. Figure 5 compares the compressive stress *vs* strain curve of aerogel *0.9* *A* in the two main planes, namely out-of-plane and in-plane. The in-plane compression, which is in direction of the pore axis, shows a very distinct elastic regime followed by a plateau and densification regime. The aerogel exhibits a yielding behavior (at around 15% strain), which is a characteristic of plastic foams^46^. A similar behavior was observed by Donius *et al*.^47^ for anisotropic nanocellulose-montmorillonite aerogels. When compressed along the axis, the pore walls elastically buckle until 15% strain and collapse when strained further resulting in a yielding and a constant stress of around 20 kPa. The out-of-plane view of SEM micrographs of the uncompressed and compressed (out-of-plane, in-plane) *0.9* *A* aerogel sample is shown in inset of Fig. 6. The samples were compressed up to 85% strain. The out-of-plane compressed image mainly shows pore walls bending at the hinges. There are a few pore walls that are cracked under the strain. The in-plane compression, in contrast, shows a complete structural collapse with loss of pore morphology. As can be seen from the inset images, out-of-plane compression allows aerogels to bounce back whereas in-plane compression causes a structural failure, when compressed up to a strain of 85%. The elastic behavior of aerogels upon out-of-plane compression can also be seen in Movie S1 and Figure S5. The compression modulus for in-plane compression is 112 kPa compared to 14 kPa for out-of-plane compression. The energy of absorption is 14 kPa while compressive strength is 35 kPa for in-plane compression, compared to 4 kPa and 22 kPa when the same aerogel is compressed out-of-plane. The densification strain is 74% for in-plane compression and 86% for out-of-plane compression. The data indicate highly anisotropic behavior of these aerogels, which can behave as an elastic or plastic foam based on the direction of property measurement.

### Arresting the gels in a non-equilibrium state

Building on the understanding that the swelling characteristics of a gel influences the morphology and mechanical properties of the resulting aerogel, we hypothesized that the swelling kinetics of a gel influences its properties. We therefore analyze the swelling kinetics of the gel and how the swelling relates to the properties of the corresponding aerogels. Figure 6a shows the swelling kinetics of the organogel for a period of 48 h when immersed in 0.9 AVF. The organogel reached a maximum swelling ratio of about 6. The values in the inset of Fig. 6a are normalized with the swelling ratio at 48 h, which indicates that the swelling reached an equilibrium value at 24 h. This further implies that any appreciable change in the aerogel properties is not expected after 24 h of swelling. Shrinking was observed when the swelled gels were immersed in pure water, which equilibrated at the volumetric swelling ratio of around 1.5–2.3 (Fig. 6b). The density and pore volume of the aerogels synthesized from these hydrogels is demonstrated in Fig. 6c. The density decreases as the solvent exchange time for the organogel increases and, consequently, the pore volume of the aerogel increases. This is explained by the swelling of the organogel, which increases as a function of time.

The compressive stress *vs* strain curves generated from out-of-plane compression of the aerogels are shown in Fig. 6d. Similar curves were observed in Fig. 4b, with short linear elastic region and a long plateau region followed by a densification region. It is noteworthy that the stress-strain curves for the aerogels obtained after 24 h of solvent exchange did not vary significantly. This is most likely due to the extremely low relative density (~0.02) and high pore volume (98%) observed for aerogels obtained after 24 h of swelling. The swelling reaches an equilibrium within 24 h and hence the corresponding aerogels from 24 and 48 h of swelling have the highest pore volume and lowest relative density, achievable by the solvent exchange approach for this polymer concentration. The SEM micrographs of the out-of-plane view for the aerogels obtained after varying solvent exchange time is shown in Figure S6. All the samples show a honeycomb pattern.

The resulting mechanical properties of the aerogels produced under different swelling conditions is listed in Table 3. It is evident from the values in the table that the properties of aerogels can be conveniently tailored by varying the time for solvent exchange. The compression modulus ranges from 13–160 kPa, absorption energy ranges from 4–35 kPa, and the compressive strength ranges from 22–151 kPa. A wide densification strain is also observed ranging from 65 to 88%. This wide latitude of values opens the opportunity to achieve a predetermined aerogel mechanical property (stiffness, toughness and strength) by the proposed solvent exchange approach.

**Table 3:** Properties of the aerogels obtained after solvent exchange for given time periods.

	4 h	12 h	24 h	48 h
Density, *ρ*_a_(g/cm^3^)	0.049	0.038	0.030	0.025
Pore Volume (%)	96.2	97.1	97.7	98.1
Relative density (*ρ*_a_/*ρ*_CA_)	0.038	0.029	0.023	0.019
Compression Modulus (kPa)	160	54	13	14
Absorption Energy (kPa)	35	10	7	4
Compressive strength (kPa)	151	40	26	22
Densification strain (%)	65	82	88	86

We suggest that the approach described in this study can be extended to other polymers or even inorganic aerogels, provided a suitable solvent is identified based on the Hansen solubility parameters. While this study is focused on freeze dried aerogels, the concept can be expected to be equally applicable for supercritical dried aerogels, upon identifying the interaction of the polymer or the inorganic compound with supercritical CO_2_ based on the HSP of supercritical fluids^48^.

## Conclusions

An innovative approach to synthesize ultralight, anisotropic aerogels with tailored mechanical properties is demonstrated. The use of the solubility parameter theory to control swelling behavior of the gels allowed a remarkable control over the final mechanical performance of the aerogels. The solvent exchange approach allowed us to synthesize aerogels with densities as low as 0.025 g/cm^3^ with no need for optimizing the precursor polymer concentration. The synthesized aerogels exhibited a wide range of stiffness (14–340 kPa), toughness (4–103 kPa), strength (22–373 kPa) and compressibility (35–87%). Additionally, a unidirectional and controlled freezing approach introduced anisotropy in the aerogels inducing both elastic and plastic deformations, depending on the loading direction. The morphological and mechanical properties of the aerogels can be further tuned by arresting the gels in a non-equilibrium state during solvent exchange. By using this approach, aerogels can be achieved with tunable stiffness (13 to 160 kPa), toughness (4 to 35 kPa), strength (22 to 151 kPa) and compressibility (65 to 88%).With impressive and tunable mechanical properties along with anisotropy, these aerogels can potentially be used as shock absorbers, in thermal and acoustic insulator, among many others.

## Methods

### Materials

Cellulose acetate (CA) flakes provided by Eastman Chemical Co. with degree of substitution of 2.45 and acetyl content of 39.7% and were used as received. Reagent grade acetone (99.5%), triethyl amine (TEA) and the cross-linking agent 1, 2, 4, 5-benzenetetracarboxylic acid (Pyromelletic Dianhydride, PMDA) were purchased from Sigma Aldrich. Deionized (DI) water with pH 6.74 was used. Liquid N_2_ cylinder was bought from Airgas (NC).

### Organogel synthesis

Cellulose acetate (CA) gels were synthesized from a homogeneous solution of 4 wt% CA in acetone was formed by stirring in a 100 ml Pyrex bottle for 24 hours. Based on the assumption that one PMDA molecule reacts with two hydroxyl groups on different CA chains, a CA: PMDA molar ratio of 2:1 is expected for complete cross-linking. In the present study a CA: PMDA molar ratio of 8:1 was used. The molecular weight of one unit of CA with degree of substitution 2.45 was calculated as 264.6 g/mol. To obtain the gel (organogel) in acetone, the CA solution was stirred with PMDA cross-linker for approximately 5 h to ensure complete dissolution. The catalyst (triethyl amine, TEA), 0.05 vol%, was added to the previous solution while stirring for another 30 s. A 10 ml of the solution was then transferred to a cylindrical mold and allowed to set into an elastic gel for 24 h.

### Solvent exchange and swelling

The organogel was cut into cuboidal shape of 1 × 0.8 × 0.5 cm. In order to investigate the effect of solvency on gel swelling, acetone volume fractions (AVF) of 1, 0.9, 0.75, 0.5, 0.25 and 0 were used as media for immersion (48 h). The effect of solvent exchange time on gel swelling behavior was accessed for organogels with 0.9 AVF; the organogels were sampled at 4, 12, 24 and 48 h intervals.

The volume of the gels was measured via volume displacement method, where the gel was carefully placed in a glass cylinder filled with known solvent and the change in height of the solvent was measured via a Vernier caliper. The volumetric swelling/shrinking is reported by normalizing the volume of the organogel at time ‘*t*’ with respect to the initial volume of the organogel.

2 $${\rm{Volumetric}}\,{\rm{Swelling}}\,{\rm{Ratio}}=\frac{{V}_{t}-{V}_{0}}{{V}_{0}}$$

where, *V*_*t*_ is volume of the gel at time ‘*t*’ and *V*_*0*_ is the initial volume.

A total of five experiments were carried out for each solvent concentration and each time interval. The average values of swelling along with the standard deviation are reported. The resulting organogels were immersed for 72 h in DI water, which was replaced every 24 h. Thereafter, the obtained gels in water are referred to as hydrogels. The gels are abbreviated as *f*A, where, ‘*f*’ is the AVF of the solvent in which the organogel was immersed. For example, *0.9* A indicates a gel that resulted from immersion of the organogel in a solvent comprising an AVF = 0.9.

### Aerogel synthesis

The respective hydrogels were frozen directionally to ensure pore alignment. This was achieved using a computer-controlled temperature base plate (Linkam LTS350) using cooling with liquid N_2_ and electrically heated coils to maintain the temperature at −80 °C. The frozen hydrogel were then transferred to a lyophilizer (Labconco FreeZone 2.5 Freeze Dryer) operating at −53 °C and 0.113 mbar, which is below the triple point of water^49^. The frozen hydrogels were dried for ~24 h to give the CA aerogel. The label given to the aerogels use an abbreviation according to the immersion solvent (solvent exchange) for the corresponding organogel. For example, an aerogel prepared after immersing organogel in 90 vol.% acetone (AVF = 0.9) is referred to as “*0.9* A”.

### Density and Pore Volume

Aerogel density (*ρ*_a_) was calculated by measuring its mass and volume. The mass was measured by an analytical balance, Fisher Scientific Accu-225D, which has least count of 0.1 mg. The volume was determined by measuring the dimensions using a digital Vernier caliper. Average density is reported after 5 measurements for 3 different aerogels. The % pore volume of the aerogels was calculated using Equation (2):

3 $$Pore\_Volume=(1-\frac{{\rho }_{a}}{{\rho }_{CDA}})\ast 100 \% ,$$

where, *ρ*_a_ is bulk density of aerogel *ρ*_CA_ is bulk density of CA flakes (1.3 g/cc)^50^.

*Turbidity measurements:* Turbidity was measured by using a Thermo Scientific turbidity meter (Orion^TM^ AQ4500). The instrument was calibrated using 5 USEPA approved primary calibration standards. The samples were measured in transmittance mode. The values are reported in turbidity units (NTU).

### Scanning Electron Microscopy (SEM)

The imaging was performed with a Field Emission Scanning Electron Microscope (FESEM), FEI Verios 460 L. The aerogels were fractured under liquid N_2_ using a sharp clean blade to image the in-pane and out-of-plane cross-sections. The samples were fixed on the metal stub using a double-sided carbon tape. The as prepared SEM samples were coated with a 5-nm layer of gold and platinum to capture secondary electrons from the surface and to reducing charging.

### Mechanical Compression testing

The compressive stress-strain profiles were obtained via Instron Series IX using a compressive load of 0.5 N that was lowered at the rate of 5 mm/min. The aerogels were compressed in-plane (parallel to freezing direction) and out-of-plane (perpendicular to freezing direction) directions. The compression modulus was obtained as the slope of initial linear region (at 1% strain). The energy of absorption (related to toughness) was calculated as the area under the curve, from 0 to 70% strain. The compressive strength was reported as the stress obtained at 70% strain and the densification strain was found as the x-intercept of the tangent from the densification region. Prior to compression testing, the aerogels were equilibrated for at least 48 h in a room under controlled relative humidity of 50%.

## Electronic supplementary material

Video S1 of Supporting Information

Supplementary Information
