# Supplementary material for: Synthesis of organic aerogels with tailorable morphology and strength by controlled solvent swelling following Hansen solubility
Source: Sci Rep. 2018 Feb 1;8:2106. doi: 10.1038/s41598-018-19720-4 (PMC5794995; doi:10.1038/s41598-018-19720-4)
Supplement: Supplementary file 2 — Supplementary Information [file 41598_2018_19720_MOESM2_ESM.pdf]

Supporting Information

Synthesis of organic aerogels with tailorable morphology and strength by controlled solvent swelling following Hansen solubility

Anurodh Tripathi[1], Gregory N. Parsons[1], Saad A. Khan[1,*], and Orlando J. Rojas[1,2,*]

[1] Department of Chemical and Biomolecular Engineering, North Carolina State University, Raleigh, NC 27695, USA.

[2] Department of Bioproducts and Biosystems, School of Chemical Engineering and Department of Applied Physics, School of Science, Aalto University, FI-00076, Espoo, Finland.

* Authors for correspondence: Orlando J. Rojas. Email: orlando.rojas@aalto.fi, Tel. +358-505124227; Saad Khan, E-mail: khan@ncsu.edu.

This Supporting Information document contains nine (9) pages with reference to one (1) video; six (6) figures and one (1) table.

Video S1 (available via web link):

Demonstration of the high compressibility of CA aerogels when compressed out-of-plane.

Hansen Solubility Parameter: The general concept that "like dissolves like" implies the use of a solvent with similar physiochemical properties as the solute [33]. The physiochemical properties of a material can be identified by its cohesive energy density (*E/V*), where '*E*' is the energy of vaporization and '*V*' is the molar volume of the material. The square root of cohesive energy density of the material is defined as Hildebrand solubility parameter (δ). If the δ of a

solvent and a solute are similar, then it is expected that they mix well. The Hildebrand solubility parameters works well for non-polar hydrocarbon systems but fail for polar solvents and solvents that exhibit hydrogen bonding. Hansen further improved upon Hildebrand solubility theory by dissociating the cohesive energy density into three components: the dispersion (δ_d), the polar (δ_p) and the hydrogen bonding (δ_h) that arise, respectively, from van der Waals, dipole and hydrogen bonding interactions. These three components, the Hansen solubility parameters (HSP), can be factored in or calculated as *Ra*, Equation 2:

$$Ra = \sqrt{4\left(\left(\delta_{d1}^2 - \delta_{d2}^2\right) + \left(\delta_{p1}^2 - \delta_{p2}^2\right) + \left(\delta_{p1}^2 - \delta_{p2}^2\right)\right)}$$

where, *Ra* is the difference between the HSP of a solvent (*1*) and a polymer (*2*). The constant 4 is from an empirical correlation. The solubility is maintained if $Ra < R_0$, defined as the "interaction radius" of the polymer, which is measured experimentally. A Relative Energy Difference (RED) is defined as Ra/R_0. A value of RED < 1 implies good solubility for the polymer in the given solvent. The value of interaction radius R_0 for a polymer is usually calculated experimentally. The solubility of the polymer is tested with various solvents of known solubility parameters (δ_d, δ_p, δ_H) and the R_0 is identified based on the polymer solubility in the corresponding solvent. **Figure S1** visually demonstrates the Hansen sphere for cellulose acetate (CA) of interaction radius R_0 (7.6 $MPa^{1/2}$). The values of the solvents (not drawn to scale) are denoted on the graph. As indicated, water lies far away from the sphere due to high δ_H (42.3 $MPa^{1/2}$), and hence is the non-solvent for CA. Acetone lies on the boundary of CA Hansen sphere, whereas a solvent blend with 90% acetone volume fraction lies inside the Hansen sphere and therefore is the best solvent for CA.

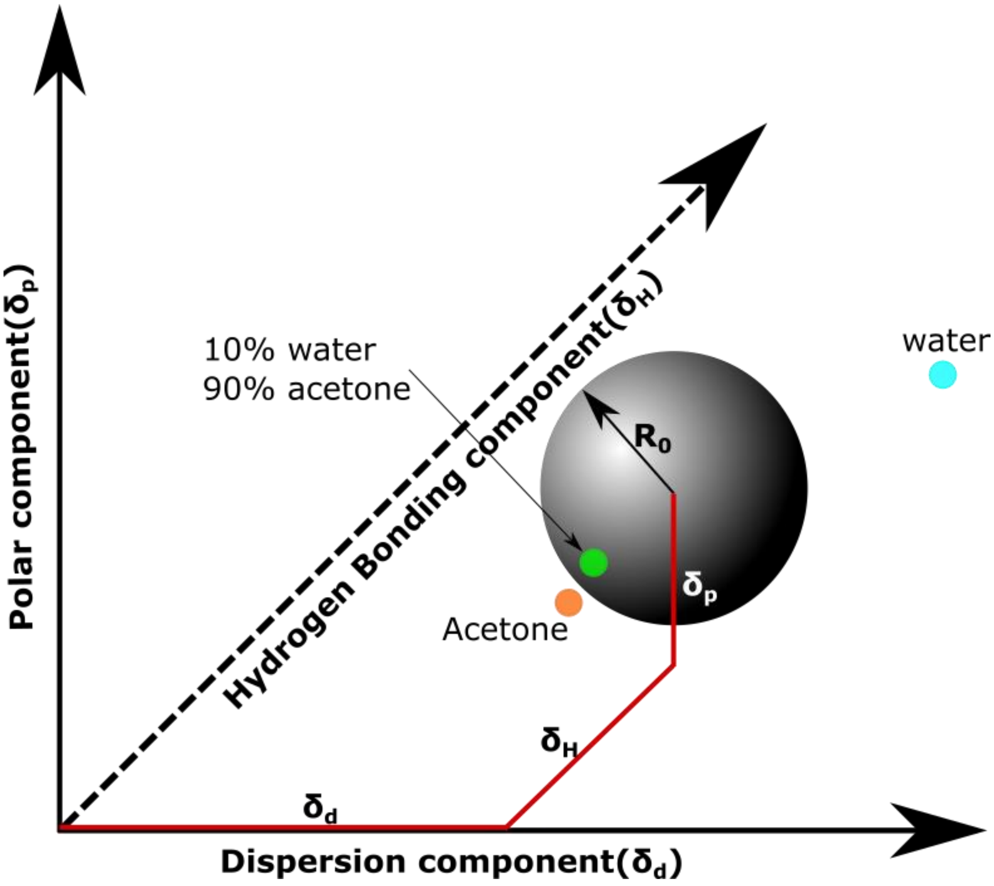

Figure S1: Visual representation of the Hansen solubility sphere along with the relative position of solvents on the three components axes. The values of the solvents on the axes are not drawn to scale.

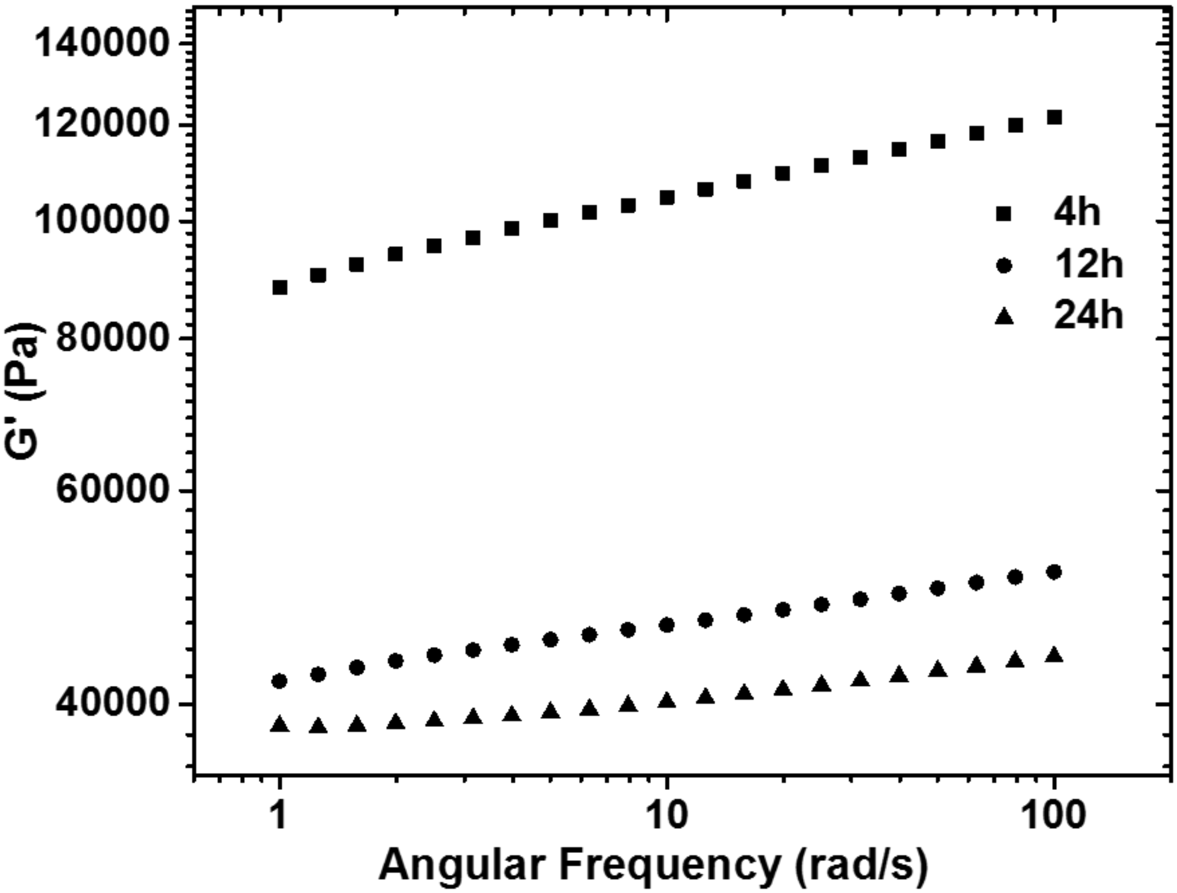

Figure S2: Frequency sweep demonstrating elastic modulus (G’) of the hydrogel obtained after solvent exchange for 4, 12 and 24 h. The organogels prepared for this measurement were 2.5cm in diameter. They were immersed in 0.9 AVF for 4, 12 and 24 h followed by water immersion for 72 h. The frequency sweep was run on a 2.5 cm diameter serrated steel flat plate geometry.

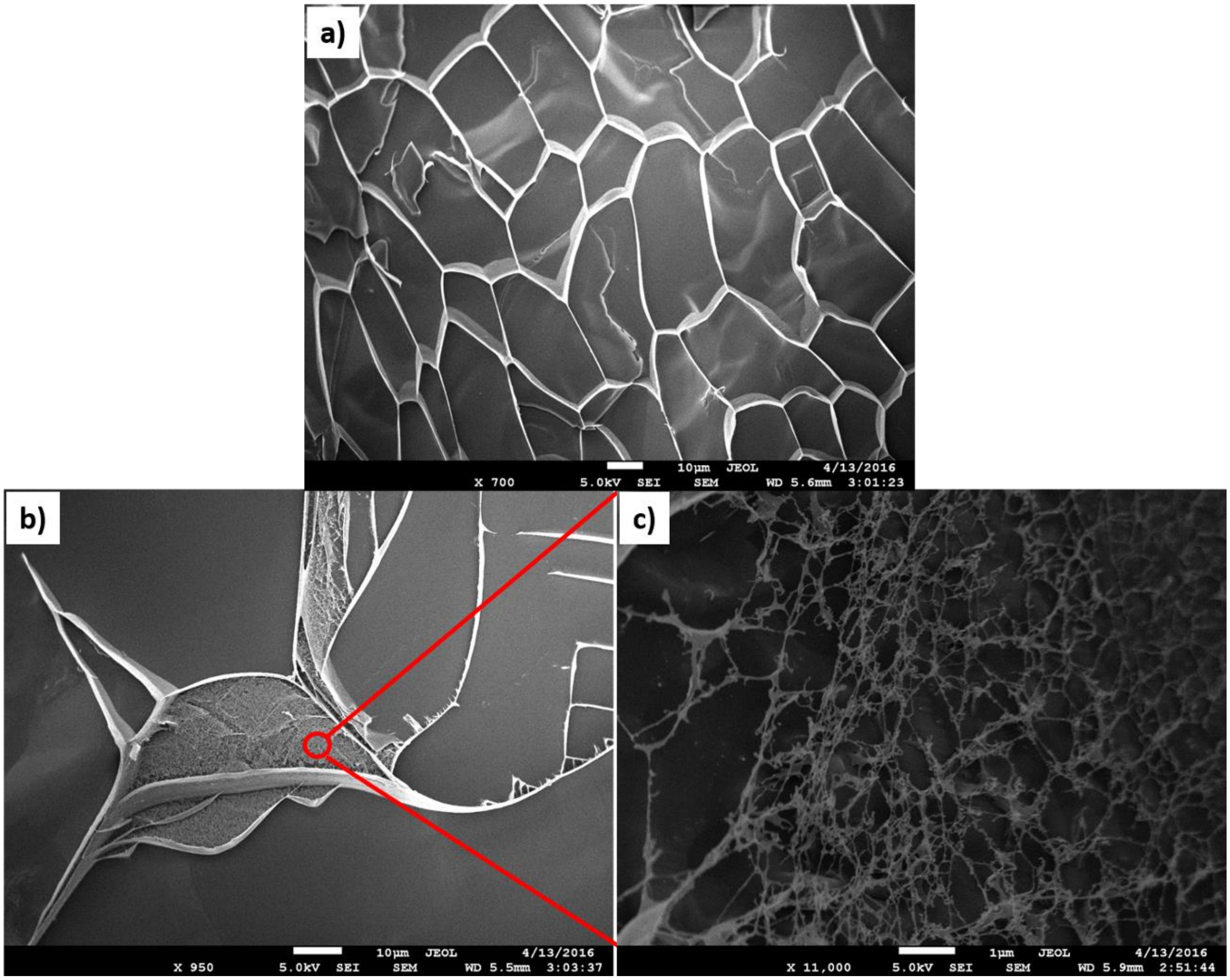

Figure S3: Cryo-SEM image of the hydrogel obtained after solvent exchange with AVF =0.9, that shows (a,b) walls of CA in honey-comb pattern , and (c) the magnification of the area where sample destruction was prevented by rapid freezing. It exhibits a phase separated CA structure.

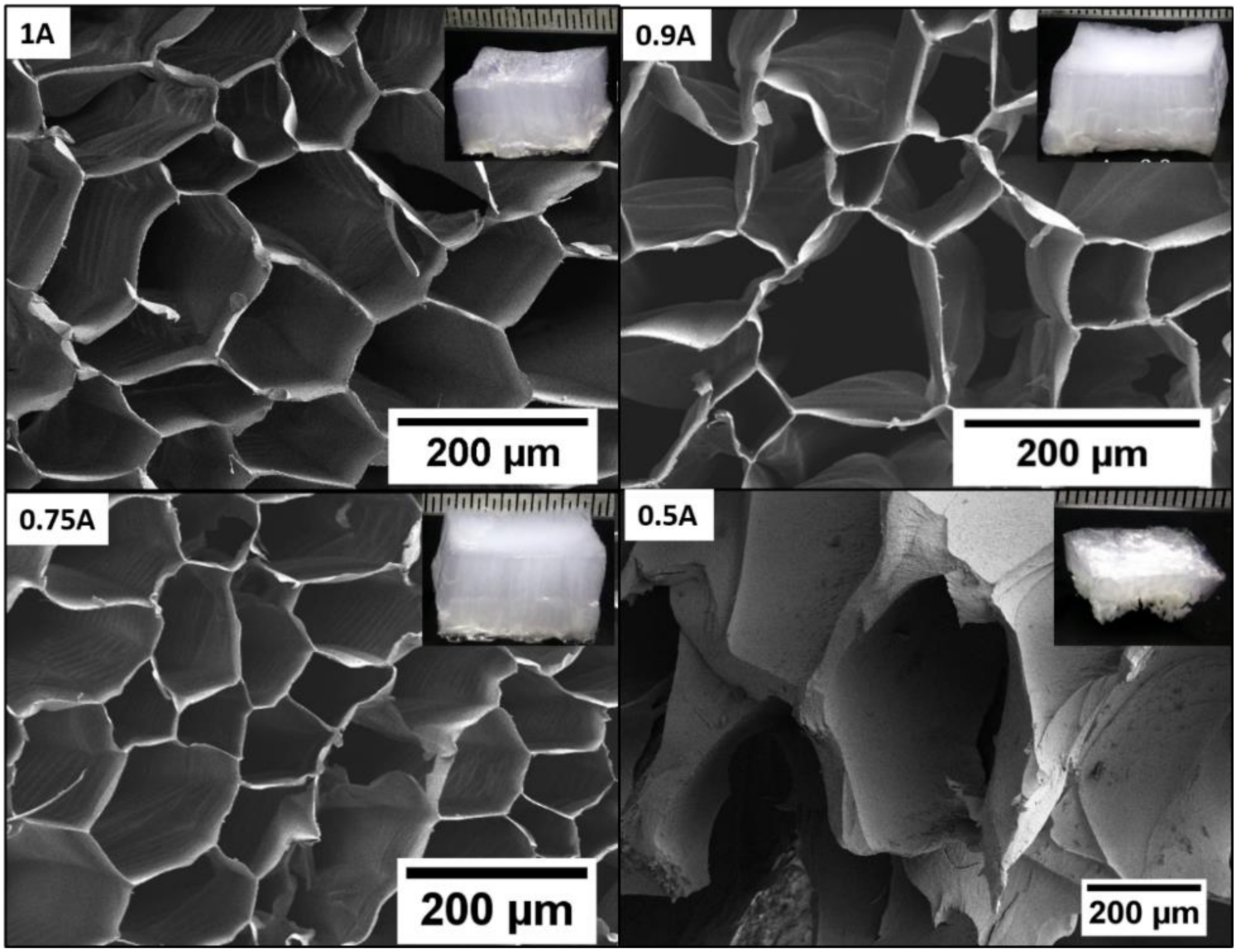

Figure S4: SEM micrographs of the out-of-plane axis of aerogels when the acetone volume fraction in solvent exchange is varied. Inset shows the camera image of the bulk aerogels

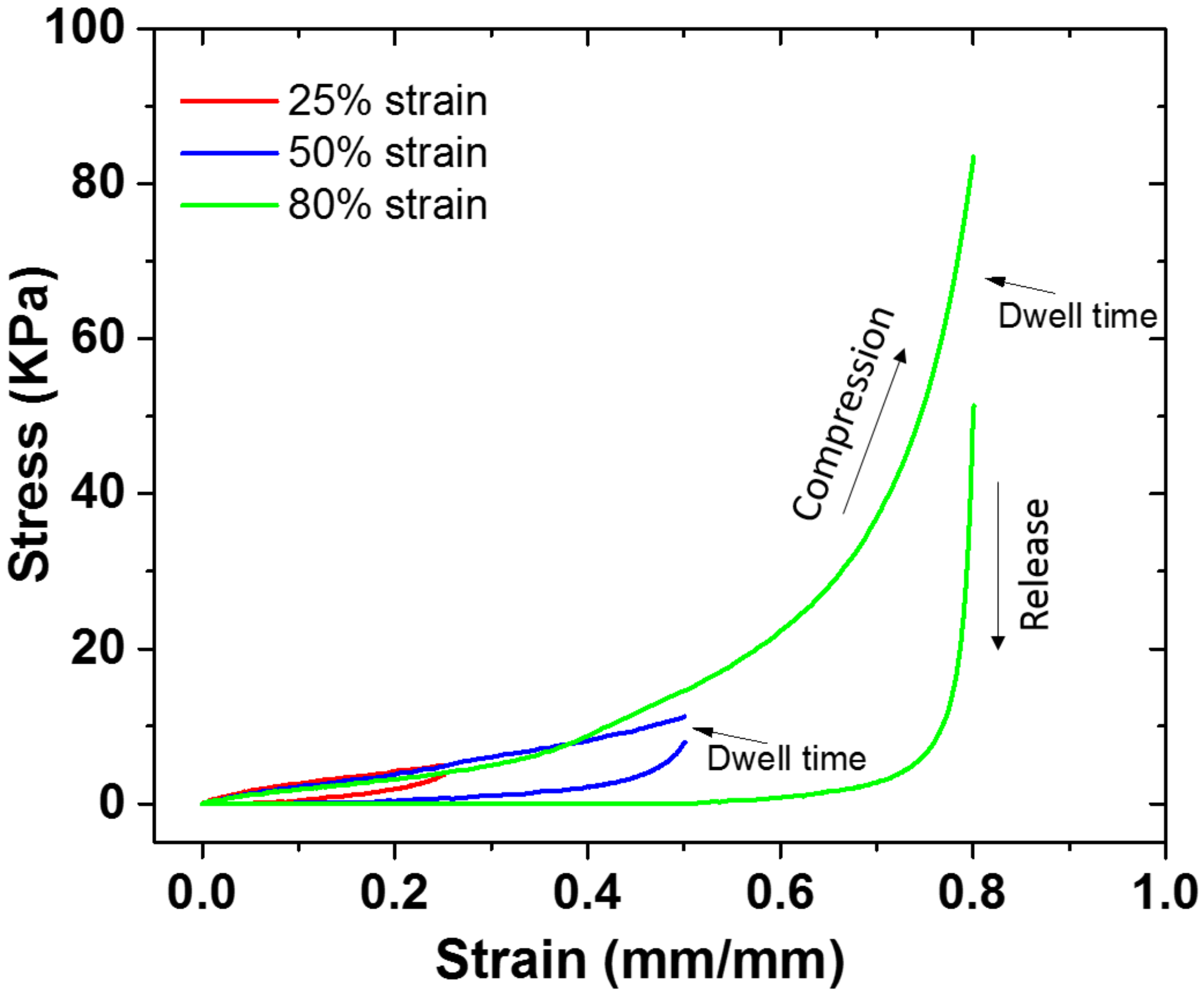

Figure S5: A cyclic compression of 0.9A aerogel when subjected to increasing strain of 25, 50 and 80%. The aerogel was kept under compression for 10 s before the release was initiated, as indicated by dwell time. The gap between the curves at maximum strain shows that the aerogel is slow to recover.

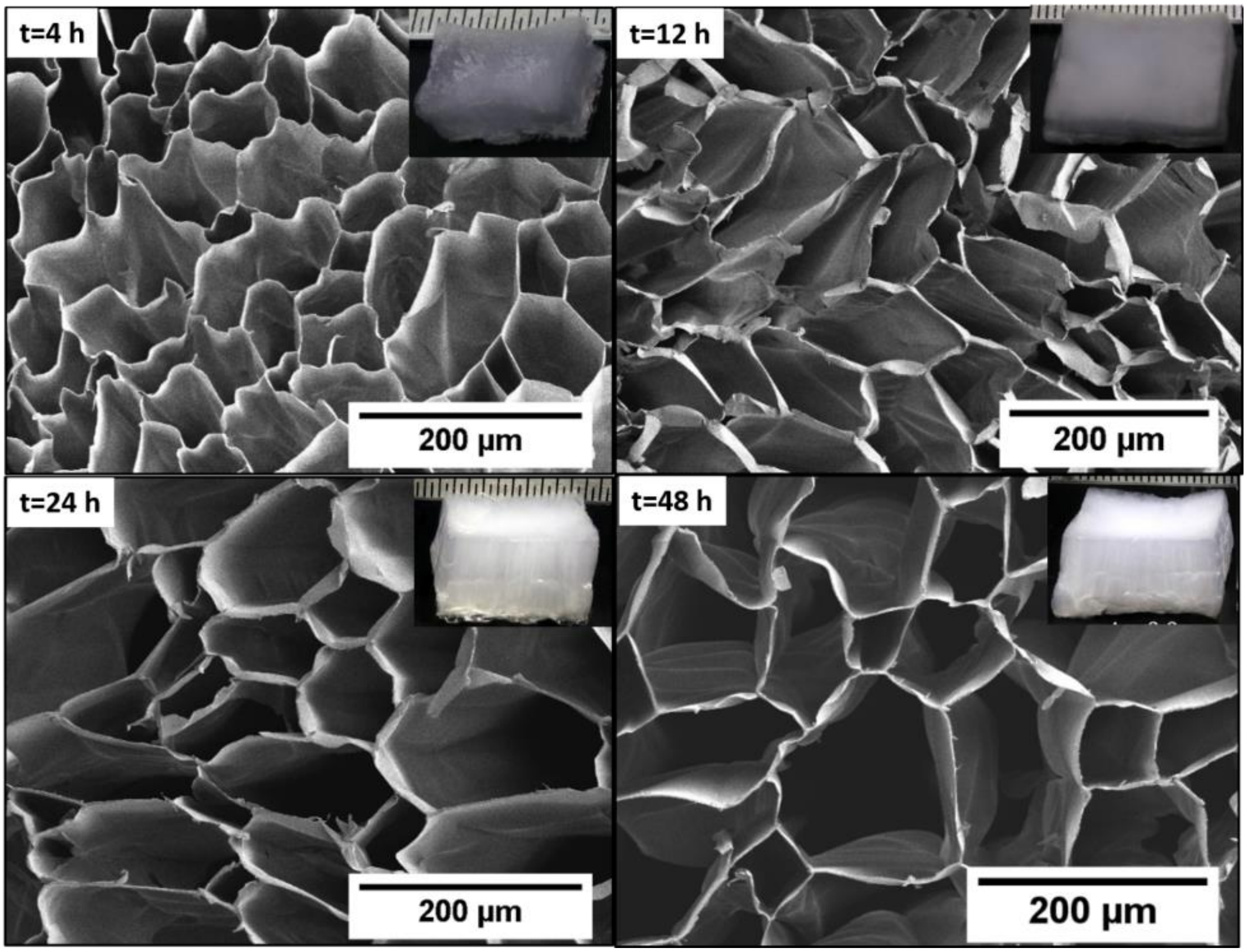

Figure S6: SEM micrographs of the out-of-plane axis of aerogels when solvent exchange time is varied. Inset shows camera image of the bulk aerogels.

Table S1: The Hansen solubility parameters of cellulose acetate and the various solvents in which it is soluble or insoluble, as indicated. The values are taken from Hansen, C. M. Hansen Solubility Parameter-User Handbook. PhD Proposal **1,** (2007)

	**δd** $MPa^{1/2}$	**δp** $MPa^{1/2}$	**δH** $MPa^{1/2}$	
**Cellulose Acetate**	18.6	12.7	11.0	
Acetone	15.5	10.4	7	Soluble
Dimethyl Acetamide (DMAc)	16.8	11.5	10.2	Soluble
Dimethyl Sulfoxide (DMSO)	18.4	16.4	10.2	Soluble
Tetrahydrofuran (THF)	16.8	5.7	8	Soluble
Dimethyl Formamide (DMF)	17.4	13.7	11.3	Soluble
Toluene	18.0	1.4	2	Insoluble
Hexane	14.9	0	0	Insoluble
Ethanol	15.8	8.8	19.4	Insoluble
Water	15.6	16	42.3	Insoluble
